# A fault diagnosis method for building electrical systems based on the combination of variational modal decomposition and new mutual dimensionless

**DOI:** 10.1038/s41598-022-27031-y

**Published:** 2023-03-20

**Authors:** Jianbin Xiong, Wenbo Qian, Jian Cen, Jianxin Li, Jie Liu, Liaohao Tang

**Affiliations:** 1 Department of the School of Automation, Guangdong Polytechnic Normal University, Guangzhou, 510665 China; 2 Guangzhou Intelligent Building Equipment Information Integration and Control Key Laboratory, Guangzhou, 510665 China; 3 Department of the School of Electronic Information, Dongguan Polytechnic, Dongguan, 523808 China

**Keywords:** Electrical and electronic engineering, Mechanical engineering

## Abstract

The fault diagnosis of building electrical systems are of great significance to the safe and stable operation of modern intelligent buildings. In this paper, it has many problems, such as various fault types, inconspicuous fault characteristics, uncertainty of fault type and mode, irregularity, unstable signal, large gap between fault data classes, small gap between classes and nonlinearity, etc. A method of building electrical system fault diagnosis based on the combination of variational mode decomposition and mutual dimensionless indictor (VMD-MDI) and quantum genetic algorithm-support vector machine (QGA-SVM) is proposed. Firstly, the method decomposes the original signal through variational modal decomposition to obtain the optimal number of Intrinsic Mode Function(IMF) containing fault feature information. Secondly, extracts the mutual dimensionless indicator for each IMF. Thirdly, the optimal penalty coefficient *C* of the support vector machine and the parameter gamma ($\gamma$) in the radial basis kernel function are selected by the quantum genetic algorithm. Finally, SVM optimized by the QGA is used to identify and classify the faults. By applying the proposed method to the experimental platform data of building electrical system, and compared with the traditional feature extraction method Empirical Mode Decomposition (EMD), Singular Value Decomposition(SVD), Local Mean Decomposition(LMD). And compared with traditional SVM, Genetic Algorithm optimized Support Vector Machine (GA-SVM), One-Dimensional Convolutional Neural Network (1DCNN) for fault classification methods. The experimental results show that the method has better effect and higher accuracy in fault diagnosis and classification of building electrical system. Its average test accuracy can reach 91.67$\%$.

## Introduction

**Figure 1:**
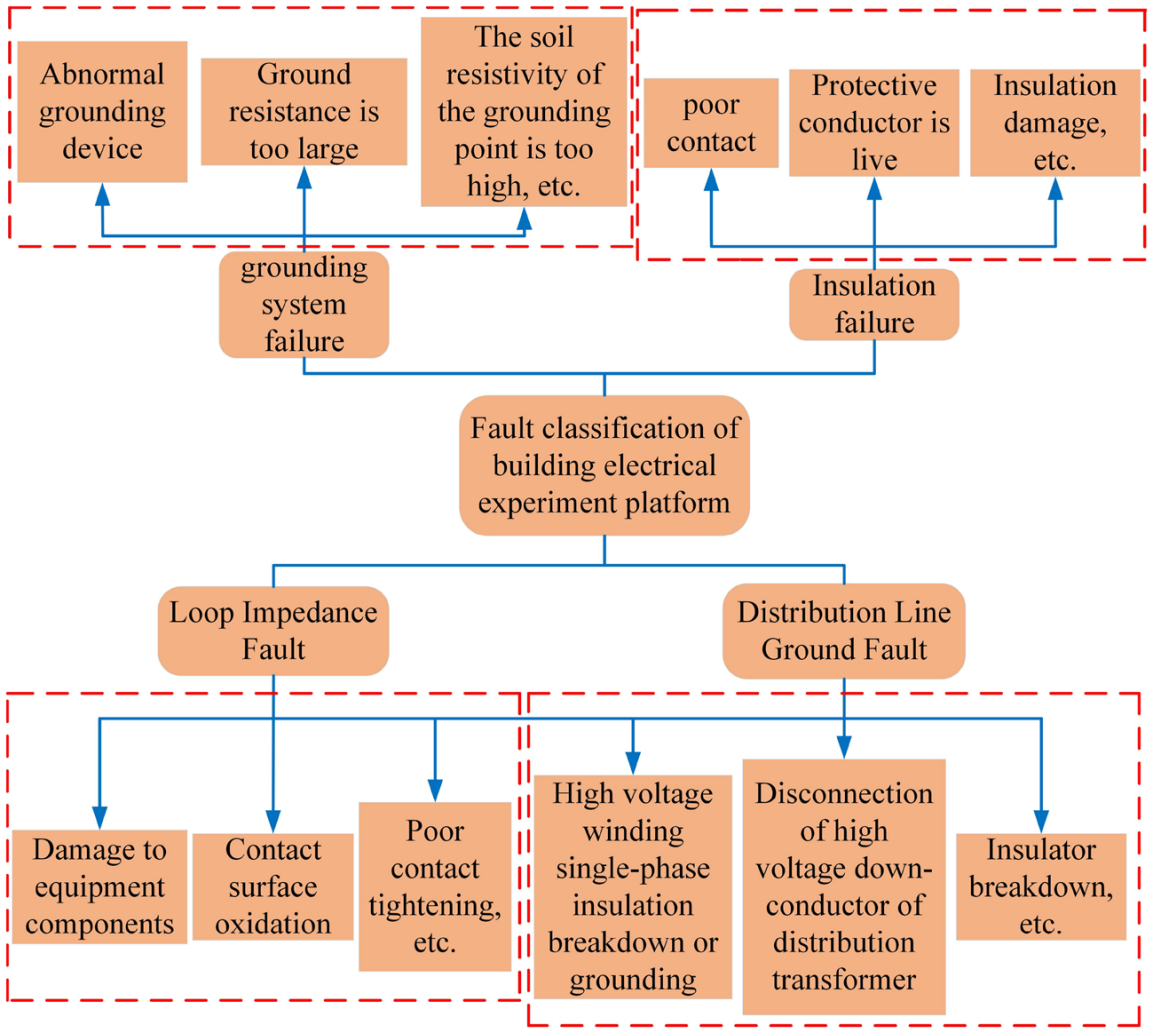
Fault classification diagram of building electrical experiment platform.

Building electrical system is a system for supervising building electricity. The normal operation of this system is crucial to the development of related industries important. Such as hospitals, airports, office buildings, etc. People are demanding more from the reliable, safe and stable operation of the electrical system of intelligent buildings. With the development of science and technology, the building electrical system will get very complicated, resulting in a wide variety of and complex failures, and the probability of failure has gradually increased. Therefore, in the face of more and more complex electrical systems, the speed and accuracy of traditional manual detection methods can no longer meet the requirements^1^. In the building electrical system, once a fault occurs, it will cause the equipment to stop working and the loss of data, and may even cause certain injuries to people^2^. It is necessary to study fault diagnosis of building electrical systems. In my country, the diagnosis technology is still in the development stage. And the intelligent fault diagnosis technology of building electrical systems started late. But its stability and efficiency have been verified, the application prospect is huge, and it has been comprehensively developed. At first, fault diagnosis of building electrical systems was checked by staff, so there would be a lot of subjectivity and uncertainty. Until now, the research of building electrical fault diagnosis at domestic and foreign is still immature^3^. In this paper, the algorithm research on feature extraction, fault classification and identification is carried out for the original resistance signal collected by the building electrical experiment platform. Figure 1 shows the fault classification diagram of the building electrical experiment platform.

In 2014, Dragomiretskiy and Zosso^4^ proposed a recent data preprocessing means VMD. It can overcome the problem of modal aliasing. In the process of decomposing, the frequency center and bandwidth of each component are determined by continuous iteration, and the signal are effectively separated^5^. Compared with EMD, the VMD method has a solid mathematical theoretical foundation and can better deal with the problem of mode aliasing. Some researchers use the VMD method for fault diagnosis of building electrical systems. For example, Achlerkar et al.^6^ proposed a single and hybrid Power Quality (PQ) disturbance detection and classification method based on VMD and decision tree for the problem of distributed grid-connected power generation system. The method has higher diagnostic accuracy and high stability. Zhang et al.^7^ raised a new approach for fault localization and fault type identification that combines VMD and Convolutional Neural Network (CNN) for the problems of complex topology, numerous branches and inaccurate fault location in distribution network. The method can accurately locate grid faults. On the basis of^8^, Wang et al.^9^ proposed a VMD and Teager–Kaiser Energy Operators (TKEOs) for the problem that high-resistance fault detection is difficult and easy to confuse capacitor switches and load switches. The High-Impedance Fault (HIF) detection approach has higher feature extraction accuracy.

However, due to the variety of fault data and the lack of obvious fault characteristics, it is far from enough to use VMD for data preprocessing. Therefore, this paper proposes to further process the decomposed data by using the extraction of mutual dimensionless indicators. The method can reduce the distance between the internal structures of each mutual dimensionless indicator, reduce the overlap of the same mutual dimensionless indicator of each fault, and can solve some problems such as various types and inconspicuous characteristics of fault data^10^. For example, Xiong et al.^11^ proposed a data preprocessing method to solve the problem of low sensitivity to fault features based on the dimensionless indicator. The experimental results proved that effectiveness of the approach. Hu et al.^12^ proposed a fault diagnosis method for a problem of low precision rate of dimensionless indicators in diagnosing nonlinear and non-stationary fault signals. It can identify fault features well and perform better fault diagnosis. Qin et al.^13^ proposed a new method about the combination of bayesian discriminant analysis and dimensionless parameter time series analysis, aiming at the problems that dimensionless indicators are not obvious for fault features and have poor ability to identify fault features. The method can effectively identify and classify faults. It can be seen from the above that the system fault data preprocessing method based on VMD-MDI is still rare. Therefore, a VMD-MDI fault data preprocessing method is proposed, which can effectively deal with problems of inconspicuous data characteristics and various types of fault data in building electrical systems.

In 1995, the concept of SVM was first proposed by Vapnik^14^. It can perform well in the case of less data, thereby reducing the amount of calculation, and its application in fault diagnosis is relatively wide. For example, Liang et al.^15^ proposed a multi-layer method based on SVM for coolers, which can be used to a small number of training samples quickly and accurately identify faults. Based on the literature^16^, Dehestani et al.^17^ proposed a semi-unsupervised fault detection method by progressively training an SVM classifier using online surveillance data, which can test unknown failures and replace the classification with these devices. Cheng et al.^18^ proposed a method for the open-circuit fault of insulated gate bipolar transistors (IGBTs) of traction inverters in catenary power supply systems. This method has a high accuracy rate for fault diagnosis of various IGBT tubes. Li et al.^19^ proposed a method combining support vector machines based on wavelet decomposition and particle swarm optimization for the problem that high-voltage circuit breakers are prone to failure. This method has high fault diagnosis accuracy. Compared with the traditional SVM, this paper proposed a SVM based on QGA optimization. It allows better selection of parameters *C*, $\gamma$ and classification of faults reaistance.

Based on the above analysis, in order to effectively extract the fault characteristic information of the resistance signal of the building electrical system and accurately diagnose and classify the fault. This paper proposed a fault diagnosis method for the building electrical system based on VMD-MDI and QGA-SVM. Firstly, the method decomposes the original feature data through VMD to obtain the optimal number of eigenmode functions (IMFs) containing fault feature information. Secondly, MDI is extracted for each IMF. Thirdly, through the optimized quantum genetic algorithm selects the optimal *C* and $\gamma$ parameters of the SVM. Finally, the QGA-SVM is used to identify and classify the faults. This method can well solve the problems of building electrical system fault diagnosis, such as various fault types, indistinct fault characteristics, and nonlinear uncertainty of fault types and modes. And compared with other methods, this method outperforms other methods. The main contributions of this paper are as follows:

1. Aiming at problems existing in traditional artificial intelligence methods, a fault diagnosis framework for building electrical systems based on VMD-MDI and QGA-SVM is proposed.
2. A new data preprocessing method combining variational mode decomposition and dimensionless index is proposed for the first time, which makes the extracted features accurately and completely express the fault information with high accuracy.
3. Based on the traditional SVM, this paper adopts QGA-SVM. This method can effectively select the optimal *C* and $\gamma$ parameters, so that the classification accuracy is higher and the effect is better.
4. At present, the field of fault diagnosis of building electrical system is few, the topic of this paper is relatively novel, but also for the future of building electrical system fault diagnosis provides a good idea.

This paper mainly discusses from the following five parts. The first part mainly describes the research status and research background of building electrical system fault diagnosis at domestic and foreign. The second part introduces the relevant theories. The third part introduces the main experimental part and the analysis of the experimental results. The fourth part discusses the method in this paper. The fifth part presents the conclusions and description of future work.

## Related theories

### Related definitions

In this paper, a fault diagnosis method based on the combination of VMD-MDI and QGA-SVM is proposed to solve the problems of unclear fault data characteristics, multiple fault types, instability and nonlinearity of building electrical system. It can not only effectively extract the features of fault data, but also perform efficient fault classification. The following will introduce the definition of VMD, mutual dimensionless theory and support vector machine optimization of quantum genetic algorithm.

#### Definition of variational modal decomposition

VMD is an adaptive and completely non recursive method of modal change and data preprocessing. It has a virtue that a number of modal decompositions can be determined. The optimal center frequency and limited bandwidth of each mode can be adaptively matched in the subsequent search and solution process. In addition, it can also achieve effective separation of Intrinsic Mode Functions (IMFs) and frequency domain division of signals. Then the effective decomposition components of the given signal are obtained. Finally, the optimal solution of the variational problem is obtained. The core idea of VMD is to construct and solve variational problems. The following is the basic principle of VMD^4,20^.

First, assuming that the original fault resistance signal *N*(*t*) is decomposed into *k* components. The corresponding constraint variational expression is shown in Eq. (1):

1 $$\begin{aligned} \begin{array}{l} \mathop {\min }\limits _{\left\{ {{u_k}} \right\} ,\left\{ {{\omega _k}} \right\} } \left\{ {\sum \limits _k {\left\| {{\partial _t}\left[ {\left( {\delta \left( t \right) + j/\pi t} \right) *{u_k}\left( t \right) } \right] {e^{ - j{\omega _k}t}}} \right\| _2^2} } \right\} \\ s.t.\sum \limits _{k = 1}^K {{u_k}} = f \end{array} \end{aligned}$$

In Eq. (1), *f* is the original input signal, *k* is the number of modal decompositions, $\left\{ {{u_k}} \right\} , \left\{ {{\omega _k}} \right\}$ corresponding to the set of *k* modal components and center frequencies after decomposing, respectively, ${\delta _t}$ is the Dirac Function, and * is the convolution operator.

Then to solve Eq. (1), the Lagrange multiplication operator $\lambda$ needs to be introduced. It can transform the constrained variational problem into an unconstrained variational problem, and the augmented Lagrange expression is obtained as shown in Eq. (2):

2 $$\begin{aligned} \begin{array}{l} L\left( {\left\{ {{u_k}} \right\} ,\left\{ {{\omega _k}} \right\} ,\lambda } \right) = \alpha \sum \limits _k {\left\| {{\partial _t}\left[ {\left( {\left( {{\delta _t}} \right) + j/\pi t} \right) *{u_k}\left( t \right) } \right] {e^{ - j{\omega _k}t}}} \right\| } _2^2 \\ \quad + \left\| {f\left( t \right) - \sum \limits _k {{u_k}\left( t \right) } } \right\| _2^2 + \left\langle {\lambda \left( t \right) ,f\left( t \right) - \sum \limits _k {{u_k}\left( t \right) } } \right\rangle \end{array} \end{aligned}$$

In the above Eq. (2), $\alpha$ is a quadratic penalty factor, which acts to reduce the interference of Gaussian noise. Then, using the Alternating Direction Method of Multipliers (ADMM) iterative algorithm combined with Parseval and Fourier equidistant transform is used to optimize each modal component and center frequency. And it searchs for the saddle point of the augmented Lagrange function, then alternately searching for ${u_k}$ , ${\omega _k}$ and $\lambda$ after the iteration. The final optimization result expression is shown below, and the detailed process is shown in Eqs. (3), (4), (5):

3 $$\begin{aligned}{} & {} \frac{{\hat{f}\left( \omega \right) - \sum \nolimits _{i/k} {{{\hat{u}}_i}\left( \omega \right) + \hat{\lambda }\left( \omega \right) /2} }}{{1 + 2\alpha {{\left( {\omega - {\omega _K}} \right) }^2}}} \rightarrow \hat{u}_k^{n + 1}\left( \omega \right) \end{aligned}$$

4 $$\begin{aligned}{} & {} \frac{{\int _0^\infty {\omega {{\left| {\hat{u}_k^{n + 1}\left( \omega \right) } \right| }^2}d\omega } }}{{\int _0^\infty {{{\left| {\hat{u}_k^{n + 1}\left( \omega \right) } \right| }^2}d\omega } }} \rightarrow \omega _k^{n + 1} \end{aligned}$$

5 $$\begin{aligned}{} & {} {\hat{\lambda }^n}\left( \omega \right) + \gamma \left( {\hat{f}\left( \omega \right) - \sum \limits _k {\hat{u}_k^{n + 1}\left( \omega \right) } } \right) \rightarrow {\hat{\lambda }^{n + 1}}\left( \omega \right) \end{aligned}$$

In the above equations, $\gamma$ is the noise tolerance, which meets the fidelity requirements of signal decomposition, and $\hat{u}_k^{n + 1}\left( \omega \right)$, ${\hat{u}_i}\left( \omega \right)$, $\hat{f}\left( \omega \right)$, and $\hat{\lambda }\left( \omega \right)$ correspond to the Fourier transforms of $u_k^{n + 1}\left( t \right) ,{u_i}\left( t \right) ,f\left( t \right)$ and $\lambda \left( t \right)$, respectively.Through the VMD method, multiple sub-signals with different center frequencies are obtained. For each sub-signal, six types of mutually dimensionless metrics can be calculated. The signal processed based on VMD is not simply based on the time-frequency domain statistical characteristics of the signal, but extracts the dynamic changes of each vibration signal at different scales.

In this paper, VMD is used to decompose the original resistance data or signal into 8 effective eigenmode functions. The specific working principle and algorithm steps^4^ of VMD are as follows :

*Step* 1: Initialize $\left\{ {{u_k}} \right\}$, $\left\{ {{\omega _k}} \right\}$, $\lambda$ and $n = 0$.

*Step* 2: Let $n = n + 1$ perform an iterative loop.

*Step* 3: Update $\left\{ {{u_k}} \right\}$ and $\left\{ {{\omega _k}} \right\}$ with Eqs. (3) and (4).

*Step* 4: Update $\lambda$ with Eq. (5).

*Step* 5: Given a precision of $\varepsilon$, if the stop condition $\sum \nolimits _k {{{\left\| {u_k^{n + 1} - u_k^n} \right\| _2^2}/ {\left\| {u_k^n} \right\| }}} _2^2 < \varepsilon$ is met, stop the loop; otherwise, go to the second step to continue the loop.

**Figure 2:**
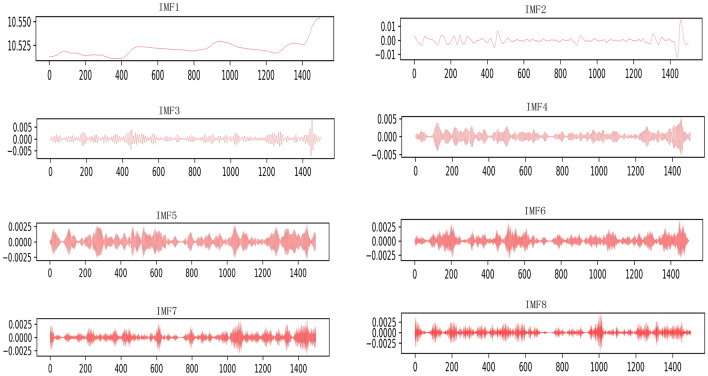
Modal diagram of VMD decomposition.

Among them, $\left\{ {{u_k}} \right\}$ , $\left\{ {{\omega _k}} \right\}$ corresponds to the set of *k* modal components and center frequencies after decomposition, $\lambda$ is the Lagrangian multiplication operator, and n is the number of iterations.In this paper, the VMD method is used to solve the problem of redundant information and noise interference in fault data. The VMD method is used to solve the problem of redundant information and noise interference in fault data, and the original building electrical system resistance fault signal is decomposed into 8 effective IMFs. This is because the center frequencies will be very similar when decomposed into 9 IMFs, resulting in modal aliasing. The effect diagram of the original signal and VMD decomposition is shown in Fig. 2 below.

#### Definition of mutually dimensionless indicator

According to the problem that dimensionless indicatores cannot effectively extract features, this paper proposed a calculation method of mutual dimensionless indicatores. This method can reduce the distance between the internal structures of each dimensionless indicator and reduce the coincidence of the same dimensionless indicator of each fault. In this paper, the mutual dimensionless indicatores and original fault resistance signal to fault signals is used as the eigenvector of the input support vector machine model to improve the accuracy of fault diagnosis. The calculation method of the mutual dimensionless indicator is as follows^11^.

Suppose there is a set of observed resistance signals *N*(*k*), and the number of *N*(*k*) is large enough. Using Eq. (6) to discretize it into $l\left( {k - {k_0}}\right) , x\left( k \right) , v\left( k \right)$ parts.

6 $$\begin{aligned} N(k) = c[l(k - {k_0}) + x(k) + v(k)] \end{aligned}$$

Make

7 $$\begin{aligned} y(k) = x(k) + v(k) \end{aligned}$$

The observed signal sample is thus defined as:

8 $$\begin{aligned} N(k) = c[l(k - {k_0}) + y(k)] \end{aligned}$$

Defining the probability density functions of random variables *s* and *y* as *p*(*s*) and *p*(*y*), respectively. The general Equation of the mutually dimensionless indicator is as shown in Table 1:

**Table 1:** Mutually dimensionless indicator equations.

Serial number	Signal ratios and indicators	Formula
1	Fault signal ratio	${X_{SFR}} = \frac{{[\int {_\Re |y{|^l}p(y)dz{]^{1/l}}} }}{{[\int {_\Re |s{|^m}p(s)dz{]^{1/m}}} }} =\frac{{\root l \of {{E(|y{|^l})}}}}{{\root m \of {{E(|s{|^m})}}}}$
2	When $l = 2,m = 1$ , the mutual waveform indicator:	${S_{SFR}} = \frac{{[\int {_\Re |y{|^\mathrm{{2}}}p(y)dz{]^{1/2}}} }}{{[\int {_\Re |s{|^{}}p(s)dz{]^{}}} }}$ $=\frac{{\root \of {{E(|y{|^2})}}}}{{E(|s|)}}$
3	When $l \rightarrow \infty ,m = 1$ , the mutual pulse indicator:	${I_{SFR}} = \mathop {\lim }\nolimits _{l \rightarrow \infty } \frac{{[\int {_\Re |y{|^l}p(y)dy{]^{1/l}}} }}{{[\int {_\Re |s{|^{}}p(s)ds{]^{}}} }}$ $=\frac{{\mathop {\lim }\nolimits _{l \rightarrow \infty } \root l \of {{E(|y{|^l})}}}}{{E|s|}}$
4	When $l \rightarrow \infty ,m = 1/2$ , the mutual margin indicator:	$C{L_{SFR}} = \mathop {\lim }\nolimits _{l \rightarrow \infty } \frac{{[\int {_\Re |y{|^l}p(y)dy{]^{1/l}}} }}{{[\int {_\Re |s{|^{1/2}}p(s)ds{]^2}} }}$ $=\frac{{\mathop {\lim }\nolimits _{l \rightarrow \infty } \root l \of {{E(|y{|^l})}}}}{{{{[E(\sqrt{|s|} )]}^2}}}$
5	When $l \rightarrow \infty ,m = 2$ , the mutual peak indicator:	${C_{SFR}} = \mathop {\lim }\nolimits _{l \rightarrow \infty } \frac{{[\int {_\Re |y{|^l}p(y)dy{]^{1/l}}} }}{{[\int {_\Re |s{|^2}p(s)ds{]^{{1/ 2}}}} }}$ $=\frac{{\mathop {\lim }\nolimits _{l \rightarrow \infty } \root l \of {{E(|y{|^l})}}}}{{\sqrt{E(|s{|^2}} )}}$
6	Directly define the mutual kurtosis indicator:	${K_{SFR}} = \frac{{\int {_\Re {y^4}p(y)dy} }}{{[\int {_\Re |s{|^2}p(s)ds{]^2}} }}$ $=\frac{{E(|y{|^4})}}{{{{[E(|s{|^2})]}^2}}}$
7	Directly define the mutual skewness indicator:	$S{K_{SFR}} = \frac{{\int _\Re {{y^3}p(y)dy} }}{{{{\left[ {\int _\Re {{\mathrm{{s}}^2}p(s)ds} } \right] }^{3/2}}}}$ $=\frac{{E({{\left| y \right| }^3})}}{{{{\left[ {E({{\left| s \right| }^2})} \right] }^{3/2}}}}$

In this paper, mutual dimensionless index extraction is used to solve the problems of a number of fault data and inconspicuous fault characteristics.

Firstly, this paper decomposes the original data signal into several effective IMF through VMD method. It can solve problems of information redundancy and noise interference in fault data. Secondly, six mutually dimensionless indicators are extracted for each IMF obtained. Finally, six dimensionless indicators with high sensitivity were selected and combined with the original resistance signal as the new input feature vector, which can effectively express the characteristics of the signal.

#### Definition of support vector machine

Traditional fault diagnosis methods are generally knowledge systems based on expert systems, so some limitations and subjectivity are inevitable. SVM^21^ is a powerful tool for solving practical and nonlinear problems based on statistical theory. It can give relatively accurate results even when the amount of available sample data is relatively limited. The original model of SVM was first published by Vapnik^22^, which belonged to a supervised learning classification algorithm, and SVM was not used to solve the fault problem until the late 1990s^16^.

The working principle of SVM is as follows. Firstly, the SVM uses a linear separation hyperplane to divide the training samples into two categories. Secondly, an optimal decision hyperplane that maximizes the boundary between the two parallel support surfaces is found. Finally, the linear classification of the SVM is used a function as well as a linear hyperplane separates the training data into two classes^23^. The steps are briefly as follows^24,25^.

Assuming that a group of building electrical system resistance fault data signals are expressed as: $\left( {{A_k},{B_k}} \right)$, $k = 1,2,\ldots ,i$, $A \in {R^n}$, $B \in \left\{ { - 1,1} \right\}$, the SVM decision hyperplane is shown in Eq. (9).

9 $$\begin{aligned} \left( {\omega \cdot A + b} \right) = 0 \end{aligned}$$

where $\omega$ is the weight vector and *b* is the bias. The optimal hyperplane is shown in Eq. (10).

10 $$\begin{aligned} \left( {\omega ' \cdot A + b'} \right) = 0 \end{aligned}$$

where $\omega '$ is the optimal weight vector and $b'$ is the optimal bias.

To find the optimal hyperplane, only the optimal weight vector $\omega '$ and the optimal bias $b'$ are needed, as shown in Eq. (11).

11 $$\begin{aligned} min\left( {\frac{1}{2}{{\left\| \omega \right\| }^2} + C\sum \limits _{i = 1}^n {{\xi _k}} } \right) \end{aligned}$$

**Figure 3:**
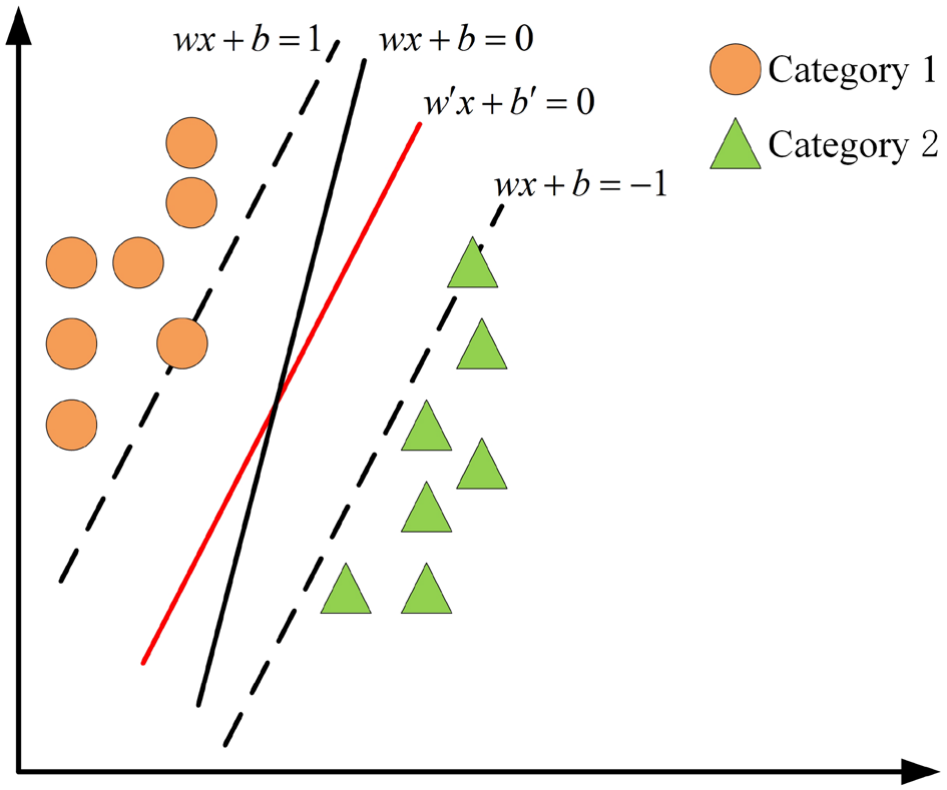
Traditional SVM classification diagram.

${B_k}\left\{ {\omega \varphi \left( {{A_k}} \right) + b} \right\} \ge 1 - {\xi _k},{\xi _k} \ge 0$ is the constraint condition. where $\xi$ is the slack variable, *C* is the penalty coefficient, and $\varphi$ is the kernel function. Figure 3 below shows the traditional SVM classification diagram, in which the black dotted line is an arbitrary plane and the category above the black dotted line represents the support vector, the black solid line is one of the arbitrary hyperplanes, and the red solid line is the optimal hyperplane.

In this paper, SVM is used for fault classification because it can handle small sample data well. Its biggest advantage is that it can perform well even with less training and testing data, thus reducing the amount of computation. The traditional SVM classifier uses the default *C* and $\gamma$ sets when solving the fault classification problem, so it is generally impossible to obtain the desired classification results. Since the difference of *C* and $\gamma$ will lead to different performance of SVM classifier, how to obtain the optimal parameters *C* and $\gamma$ is the key to improve the performance of SVM.

#### Definition of quantum genetic algorithm

QGA is based on the concept and principle of the superposition of quantum bits and quantum states. It is a newly developed probabilistic evolution method. It uses quantum revolving gates to realize the evolution of chromosomes. And compared with the traditional genetic algorithm, it can get better results^26^.

Genetic Algorithm (GA) has many iterations, slow convergence speed. And it is easy to fall into local extremum due to improper selection, crossover or mutation. In quantum computing, quantum state is used as the basic information unit, and some defects of genetic algorithm can be solved by using the characteristics. It has the advantages of small group size, large search space, fast convergence speed and so on^27–29^. In QGA, qubits are used to represent genes on chromosomes, and the evolution of chromosomes can be carried out using revolving gates. The following is an introduction to qubit encoding and quantum revolving gates.

*Quantum bit coding*

In a two-state quantum system computer, the physical medium that acts as the smallest unit of information storage is called a qubit. The difference between a quantum bit and a classical bit is that it can be any superposition state of “0” state and “1” state. Therefore, its various states are described as Eq. (12)^30^.

12 $$\begin{aligned} \left| \psi \right\rangle = \alpha \left| 0 \right\rangle + \beta \left| 1 \right\rangle \end{aligned}$$

Among them, $\left| 0 \right\rangle$ and $\left| 1 \right\rangle$ represent the spin-down state and spin-up state, respectively. $\alpha$ and $\beta$ are two amplitude constants. And it satisfied the state normalization condition as shown in Eq. (13).

13 $$\begin{aligned} {\left| \alpha \right| ^2} + {\left| \beta \right| ^2} = 1 \end{aligned}$$

where ${\left| \alpha \right| ^2}$ and ${\left| \beta \right| ^2}$ represent the probability amplitudes of the “0“ state and the “1“ state, respectively. Different from other coding schemes, QGA is a qubit based coding. Assuming that one qubit is defined with a probability amplitude of ${\left[ {\alpha \beta } \right] ^T}$, when n qubits are defined, its probability amplitude is $$\left[ \begin{array}{l} {\alpha _1}{\alpha _2}\ldots {\alpha _n}\\ {\beta _1}{\beta _2}\ldots {\beta _n} \end{array} \right]$$. The use of quantum bit encoding enables a chromosome to express the superposition of multiple states at the same time, which makes the quantum genetic algorithm have better diversity characteristics than the classical genetic algorithm.

*Quantum revolving gate* Quantum Revolving Gate (QRG) is a commonly used method in logic gates. The revolving gate can be used to update the probability amplitude of qubits so that it can achieve genetic mutation. The definition of QRG is shown in Eq. (14).

14 $$\begin{aligned} L\left( \theta \right) = \left( \begin{array}{l} \cos {\theta _n} - \sin {\theta _n}\\ \sin {\theta _n}\cos {\theta _n} \end{array} \right) \end{aligned}$$

The update process of the QRG is shown in Eq. (15).

15 $$\begin{aligned} \left( \begin{array}{l} {\alpha _n}^\prime \\ {\beta _n}^\prime \end{array} \right) = {L_\theta }\left( \begin{array}{l} {\alpha _n}\\ {\beta _n} \end{array} \right) = \left( \begin{array}{l} \cos {\theta _n} - \sin {\theta _n}\\ \sin {\theta _n}\cos {\theta _n} \end{array} \right) \left( \begin{array}{l} {\alpha _n}\\ {\beta _n} \end{array} \right) \end{aligned}$$

where $\theta$ is the angle of quantum rotation, which is used to determine the convergence rate.

This paper adopts an improved SVM, which adds an optimization algorithm Quantum Genetic Algorithm (QGA) on the basis of traditional SVM. QGA optimizes SVM in the following steps^31,32^.

*Step* 1: Initialize the population number $L\left( {{t_0}} \right)$ and set some basic parameters, including the initial population $L\left( t \right)$, the maximum evolutionary generation *M*, the maximum number *S* of the population, the binary digits *N* of each variable, and the variation ranges $\left[ {{c_{\min }},{c_{\max }}} \right]$ and $\left[ {{g_{\min }},{g_{\max }}} \right]$ of parameters *C*, $\gamma$.

*Step* 2: Measure $L\left( {{t_0}} \right)$ all individuals.

*Step* 3: The fitness values and optimal individual values of $L\left( {{t_0}} \right)$ were calculated and recorded.

*Step* 4: If the termination condition is met, exit, otherwise, continue to calculate.

*Step* 5: Measure $L\left( t \right)$ all individuals.

*Step* 6: Use the quantum revolving gate to update and evolve to obtain a new population $L\left( {t + 1} \right)$.

*Step* 7: Record the optimal individual value and fitness value.

*Step* 8: Increment the number of iterations t by 1 and return to *Step* 4.

**Figure 4:**
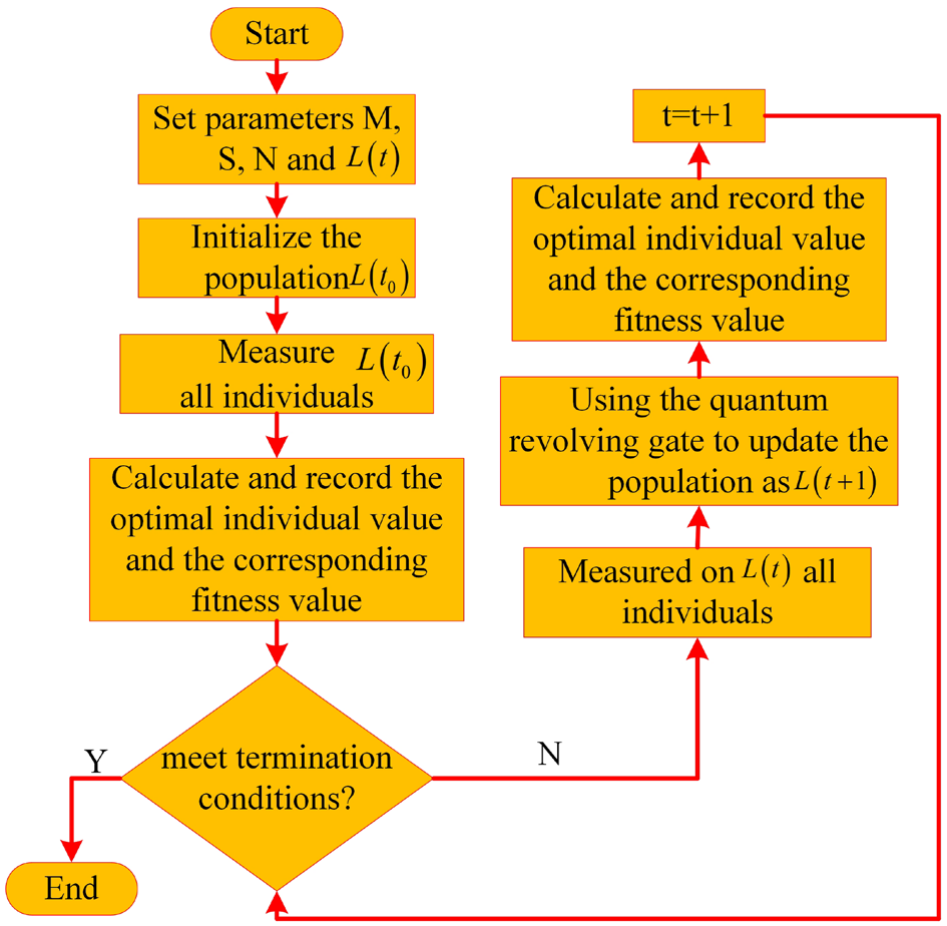
QGA optimized SVM parameter diagram.

The corresponding flowchart is shown in Fig. 4 below.

In the paper, QGA is used to deal with the problem that SVM classifier will have low classification performance due to the non-standard selection of *C* and $\gamma$ parameters. By calculating the relationship between each parameter value and fitness value, the optimization algorithm searches in the solution space to search for parameter with the maximum fitness value, which is the optimal parameter in the model.

**Figure 5:**
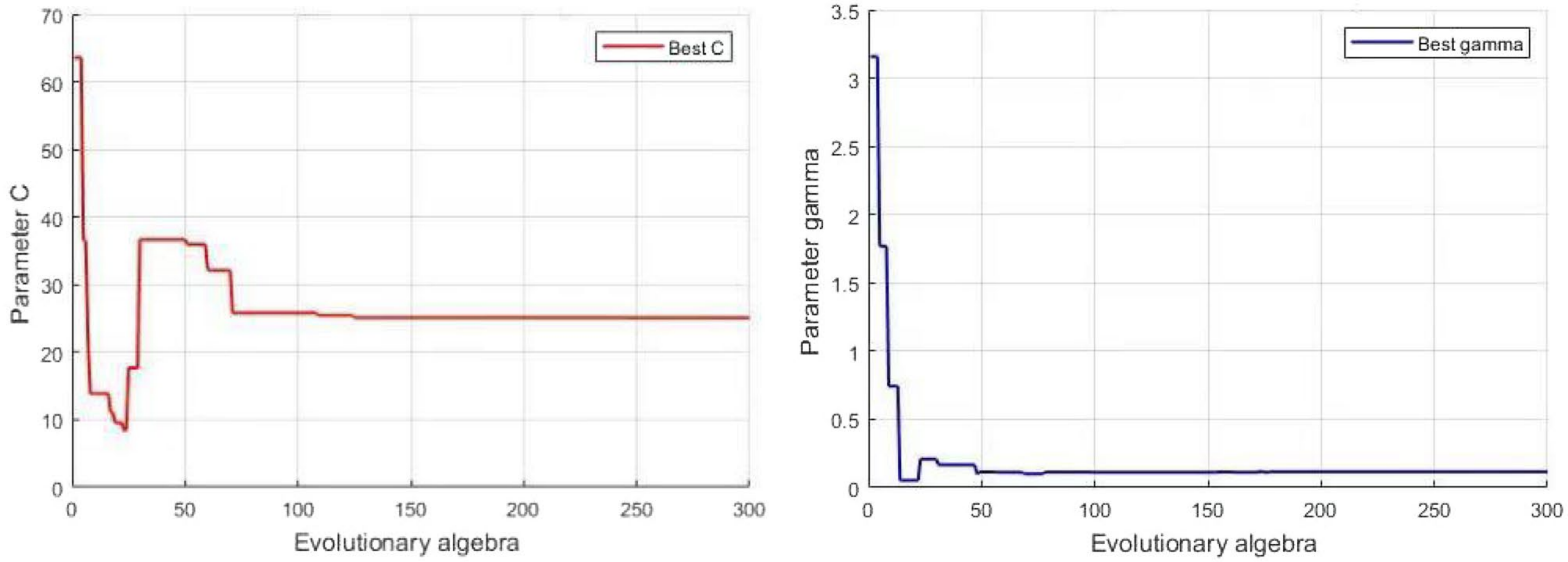
Optimization curve for parameter *C* (left) and Optimization curve for parameter $\gamma$ (right).

As shown in Fig. 5 below, when the number of iterations is 125, *C* is 25.0702. When the number of iterations exceeds 125, the parameter value of *C* remains unchanged, so it is the optimal value. When the number of iterations is 178, $\gamma$ is 0.01163. When the number of iterations exceeds 178, the parameter value of $\gamma$ remains unchanged, so it is the optimal value.

#### Construction of fusion method model based on VMD-MDI and QGA-SVM

This model is divided into three parts. It includes data acquisition, data preprocessing and fault classification algorithm. Data acquisition is the basis. The data used is the resistance value of the experimental platform collected online in real time. The platform collects 30 sets of each fault, and each set contains 50 fault resistance values.

**Figure 6:**
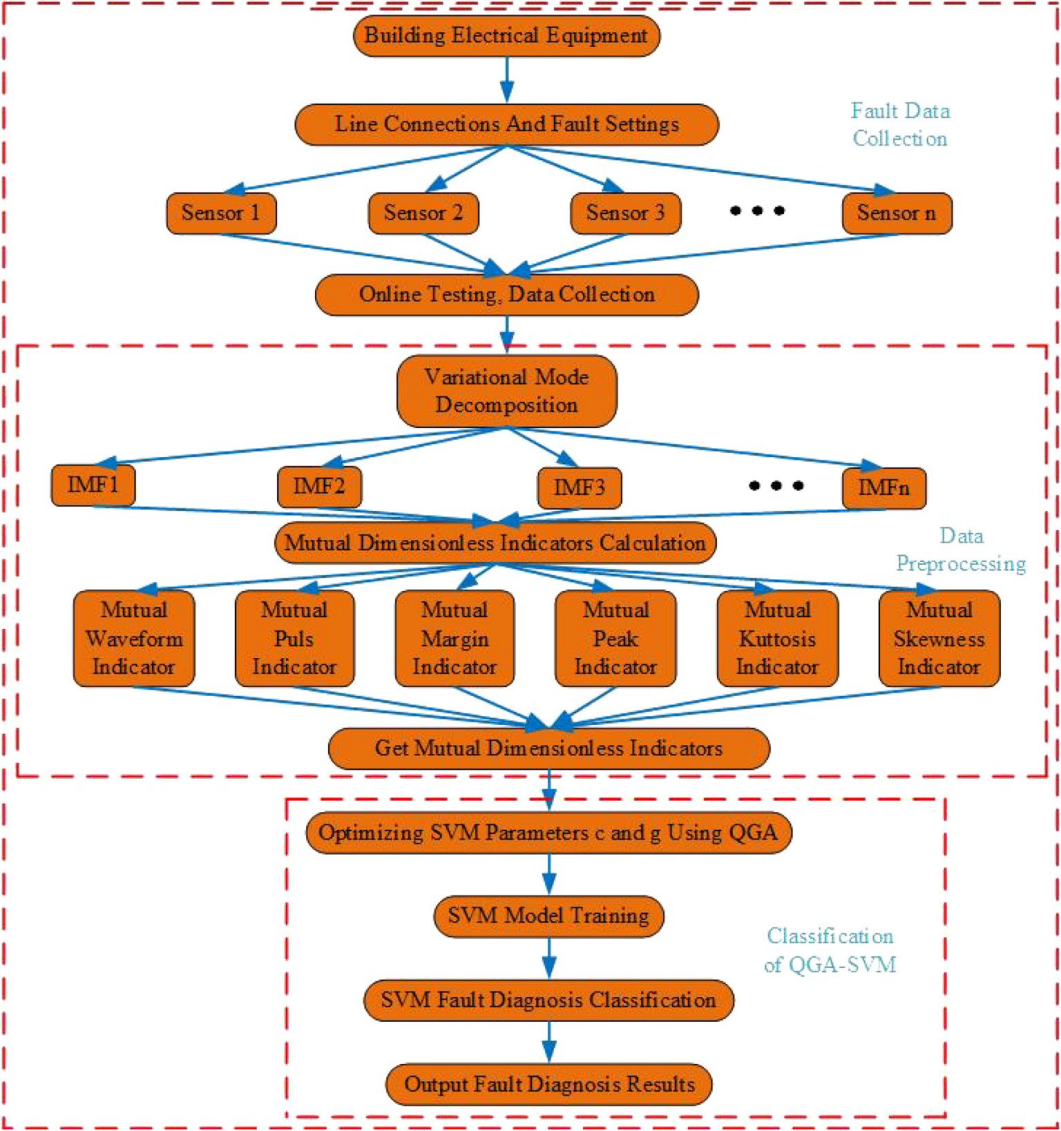
Fault diagnosis framework of building electrical system based on VMD-MDI and QGA-SVM.

The data preprocessing and fault classification algorithms are as follows. Firstly, the original fault resistance value is processed by VMD to get the optimal number of eigenmode functions (IMFs) containing fault feature information. Secondly, MDI is extracted for each IMF. Thirdly, it is verified by experiments that MDI is more sensitive to the original resistance signal characteristics, and it is selected as the input feature vector. Finally, the optimal *C* and $\gamma$ parameters of the SVM are selected by QGA. And the fault identification and classification is carried out by QGA-SVM. Its average classification accuracy reaches of 91.67percent. The specific frame diagram is shown in Fig. 6.

## Introduction of experimental equipment and analysis of experimental results

This section will focus on describing the experimental setup for measuring the fault data of the resistance signal and the instruments for collecting the data, as well as the description of the entire experimental process and the analysis of the experimental results.

### Introduction of experimental equipment

**Figure 7:**
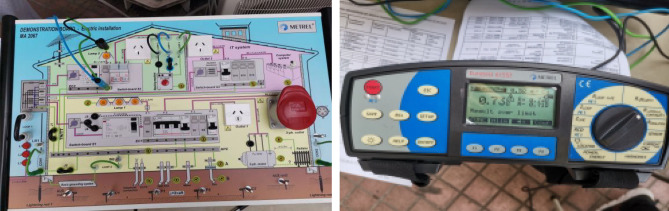
German Meicui MA2067 building electrical experimental platform (left) and Eurotest 61557 data acquisition device (right).

The software used in this experiment includes Matlab2019 and Python3.7, and the experimental devices used include building electrical experiment platform plate MA2067, Eurotest 61577, etc, as shown in Fig. 7.

The simulated faults of this experimental platform are divided into four categories: loop impedance faults, distribution line ground faults, grounding system faults and insulation faults. Each major category is further divided into several subcategories, and the experiment done in this paper is to select typical six subcategories of faults.

The setting of this fault is mainly controlled by the fault switch inside the building electrical experiment platform. There are 22 fault switches, S1–S22 respectively, and together with 53 gypsum resistors and capacitors, they form a fault setting circuit board. The fault switch can realize the connection and disconnection of different circuits and electrical equipment, thereby changing the composition of the measured object in the test circuit, and then simulating the common circuit and electrical equipment faults of the building electrical system.

### Experimental steps

*Step* 1: Select three types of six faults on the building electrical experimental platform for classification and diagnosis, namely loop impedance fault, distribution line ground fault, and grounding system fault.

*Step* 2: Connect the lines on the building electrical test platform for fault testing.

*Step* 3: Use the Eurotest 61557 data collector to collect fault data, collect 30 sets of data each time, each set of data contains 50 data points, and collect a total of 180 sets of six fault types.

*Step* 4: Read the collected data into Python, and use Python to write a VMD program to modal decomposition of the original fault resistance value, store the optimal modes of the decomposition.

*Step* 5: The modal numbers decomposed in *Step* 4 are analyzed one by one using the mutual dimensionless theory to carry out six mutual infinitesimal indices: mutual waveform indicator, mutual impulse indicator, mutual margin indicator, mutual peak indicator, mutual kurtosis indicator, and mutual skewness indicator. Extract the mutual dimensionless indicator, store the result.

*Step* 6: Use QGA to optimize the parameter penalty factors *C* and $\gamma$ of SVM.

*Step* 7: Select the mutual dimensionless index and the original resistance signal to form a new feature vector, input it to QGA-SVM for classification, and get the classification result.

### Experimental process and analysis of results

In this experiment, the data collection instrument Eurotest 61577 is used to obtain six kinds of fault data of S1, S2, S5, S8, S12 and S13 respectively. Raw data was collected at a sampling frequency of 256 Hz. Then, under the six fault conditions of S1, S2, S5, S8, S12, and S13, a total of 180 sets of raw data were collected, 30 sets of each type of fault data, and each set of data was 50 sample points. When performing fault diagnosis, the dataset is divided into training dataset and test dataset in a ratio of 3:1.

In this paper, five fault states of the building electrical experiment platform are considered, including S1 and S2 in loop impedance faults, S5 and S8 in distribution line grounding faults, and S12 and S13 in grounding system faults, as shown in Table 2. The experiment is to collect data by connecting the lines after setting each fault. By collecting the above six fault states for data preprocessing, denoising can be effectively performed, and problems such as large inter-class gap, small intra-class gap and inconspicuous fault characteristics can be solved.

**Table 2:** Brief introduction of fault settings and classification.

Fault switch	Fault category	Test point	Fault setting conditions	Number of dataset groups	Sampling frequency (HZ)
S1	Loop impedance fault	Fault between phase line L3 and line N of three-phase socket	F1 300 mA disconnected, F2 fuse disconnected	30	256
S2	Loop impedance fault	Fault between L1 phase line and N line of single-phase socket No. 2	F1 300 mA disconnected, F7 fuse disconnected	30	256
S5	Distribution line ground fault	Fault between EC2 and PE line of lamp 2	–	30	256
S8	Distribution line ground fault	Fault between phase line L1 and PE line of three-phase socket	J1, J2, J3, CON1, CON2 are inserted, F2 fuse is	30	256
S12	Grounding system failure	Basic grounding system failure	Pull out J1 and J2	30	256
S13	Grounding system failure	Lightning rod failure	CON1 is inserted, J2, CON2 are pulled out	30	256

In order to make a better diagnosis of the above-mentioned building electrical system fault data, it requires input of suitable feature vectors. The input feature vector should contain key information for each fault state.

Since the collected raw data or signals contain a lot of noise and redundant information, and the SVM model can only diagnose small sample data, it is impossible to directly input the raw data or signals into the SVM model for diagnosis. This is because the SVM model processing large data samples will make the training and testing time very long, and the effect will be poor.

Therefore, in order to solve this problem, the main steps are as follows. Firstly, using VMD to denoise and decompose to obtain the optimal modal number. Secondly, using mutual dimensionless theory to extract the mutual dimensionless index in each modal data or signal after decomposition. Thirdly, the obtained mutual dimensionless indicators are input into the QGA-SVM model for further implementation. Finally, the experimental results show that the method is different from the traditional feature extraction methods EMD, LMD, SVD and traditional fault classification methods SVM, GA-SVM, 1DCNN. This method can be well applied to fault diagnosis and classification of building electrical systems, and has a relatively high accuracy rate, which can reach of 91.67 percent.

#### How to choose the number of modes for VMD decomposition

**Figure 8:**
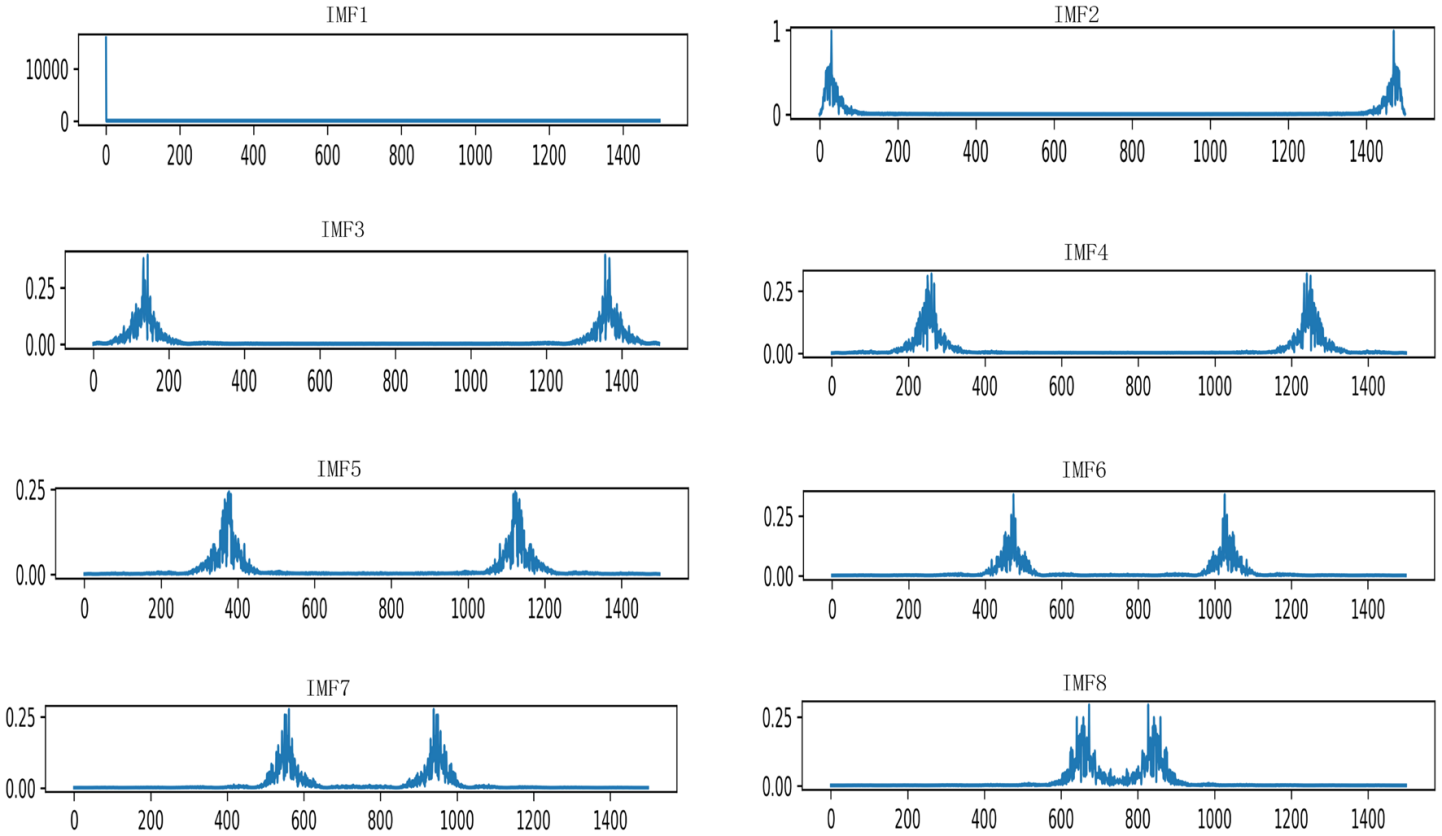
The center frequency diagram decomposed into 8 IMFs.

**Figure 9:**
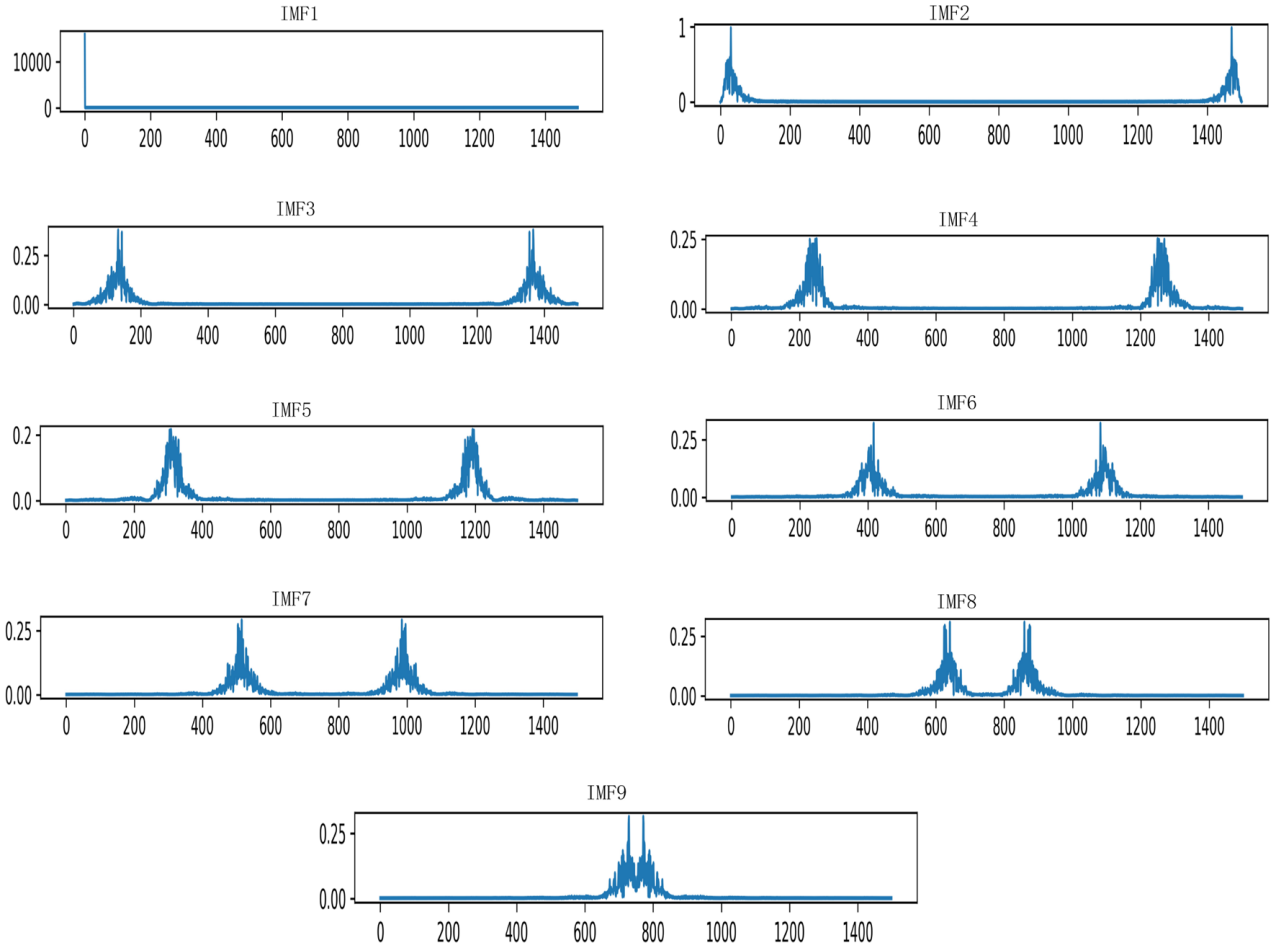
The center frequency diagram decomposed into 9 IMFs.

The original resistance value is decomposed by VMD. And different decomposition numbers have a big impact on the result of VMD, which will further affect the result of fault diagnosis. Therefore, how to choose the number of modes for VMD decomposition is a problem. If the number of decomposed modes is small, some important information will be filtered out and lost. However, when the number of decomposed modes is large, the frequency centers of adjacent modal components will be close to each other, resulting in frequency aliasing. The number of modes selected in this paper is 8. This is because when the number of modes is 9, the mode aliasing may occur. As shown in Figs. 8 and 9 below, the number of modes is 8 and 9 center frequency plots.

#### Effectiveness of VMD

**Figure 10:**
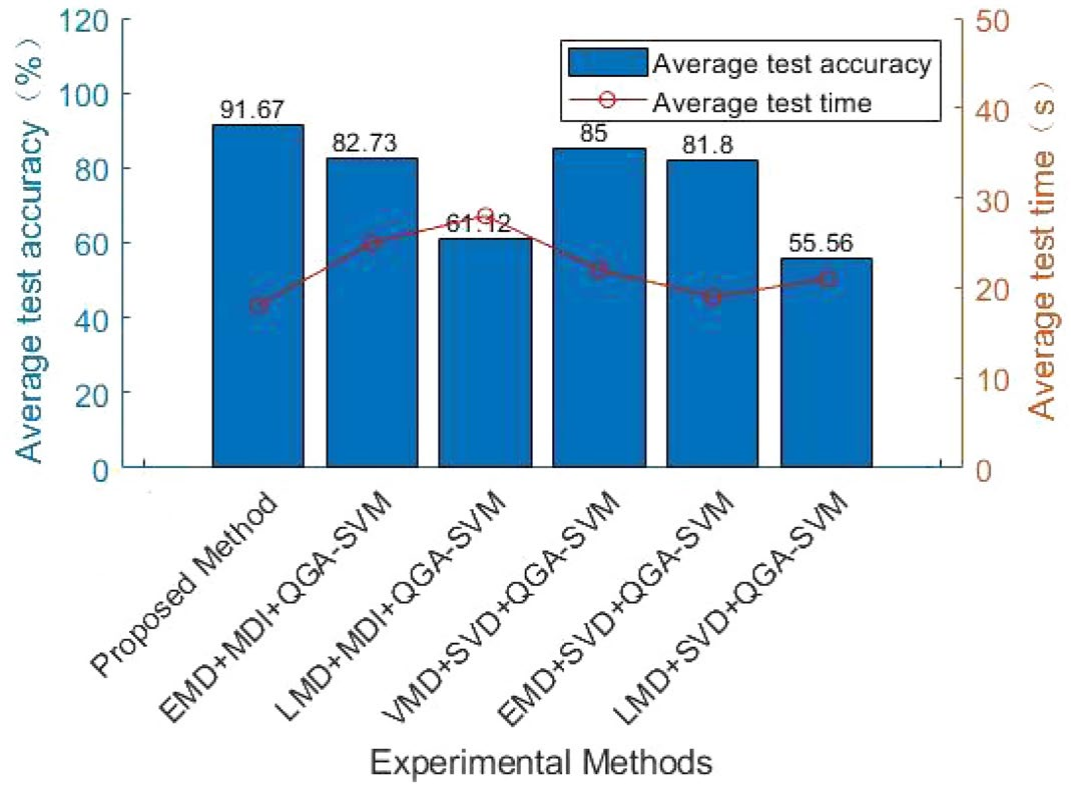
Comparison of different feature extraction methods.

In the paper, VMD is used to decompose the original fault resistance value, which can effectively denoise and extract some important fault feature information. In order to prove the effectiveness of VMD, the feature extraction method proposed is compared with EMD, LMD and SVD. The experimental results are shown in the Table 3 and Fig. 10. Table 3 and Fig. 10 shows that the denoising effect of VMD is better. VMD effectively solves the problem of modal aliasing and improves the accuracy of fault diagnosis. Its average accuracy rate reaches of 91.67%, and the running time is 18 s.

**Table 3:** Comparison of different feature extraction methods.

Kind of experiment	Experimental method	Average testing time (s)	Average test accuracy ($\%$)
1	Proposed method	18	91.67
2	EMD+MDI+ QGA-SVM	25	82.73
3	LMD+MDI+ QGA-SVM	28	61.12
4	VMD+SVD+ QGA-SVM	22	85
5	EMD+SVD+ QGA-SVM	19	81.8
6	LMD+SVD+ QGA-SVM	21	55.56

In addition, the modal decomposition number of VMD and the proportion of training data set and test data set will also have a serious impact on the experimental results. The following table shows the influence of different decomposition mode number *K* and the proportion of different data sets on the experimental results. As can be seen from the Table 4, when the number of decomposition modes is 8 and the ratio of training set to test set data is 3:1, the fault classification effect of building electrical system is the best and the accuracy is the highest, which can reach 91.67%. In addition, when the modal number is less than or greater than 8, the classification effect will be relatively poor and the classification accuracy will be relatively reduced. Therefore, IMF with decomposition mode number of 8 is selected in this paper for further experimental research.

**Table 4:** Experimental results when the decomposition number is different from the ratio of the data set.

Number of decomposition	Ratio of data sets	Average test accuracy ($\%$)
4	1:1	58.33
	2:1	62.5
	3:1	76.66
5	1:1	66.67
	2:1	75
	3:1	81.25
6	1:1	71.12
	2:1	65.84
	3:1	77.78
7	1:1	72.38
	2:1	79.99
	3:1	79.99
8	1:1	79.58
	2:1	85
	3:1	91.67
9	1:1	74.81
	2:1	75.56
	3:1	79.99

**Table 5:** Comparison of different fault classification methods.

Kind of experiment	Experimental method	Average testing time (s)	Average test accuracy ($\%$)
1	Proposed method	18	91.67
2	VMD+MDI+SVM	25	83.33
3	VMD+MDI+GA-SVM	20	88.33
4	VMD+MDI+1DCNN	10	87.5

**Table 6:** Comparison of the proposed method with other methods in the literature.

Kind of experiment	Experimental method	Average testing time (s)	Average test accuracy ($\%$)
1	Proposed method	18	91.67
2	EMD+SVD+SVM^33^	23	72.73
3	1DCNN^34,35^	17	67.56

#### Effectiveness of QGA-SVM

**Figure 11:**
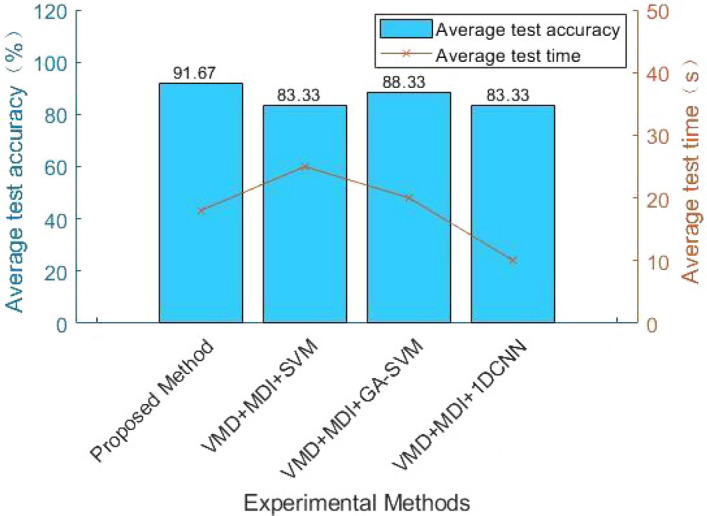
Comparison of different fault classification methods.

**Figure 12:**
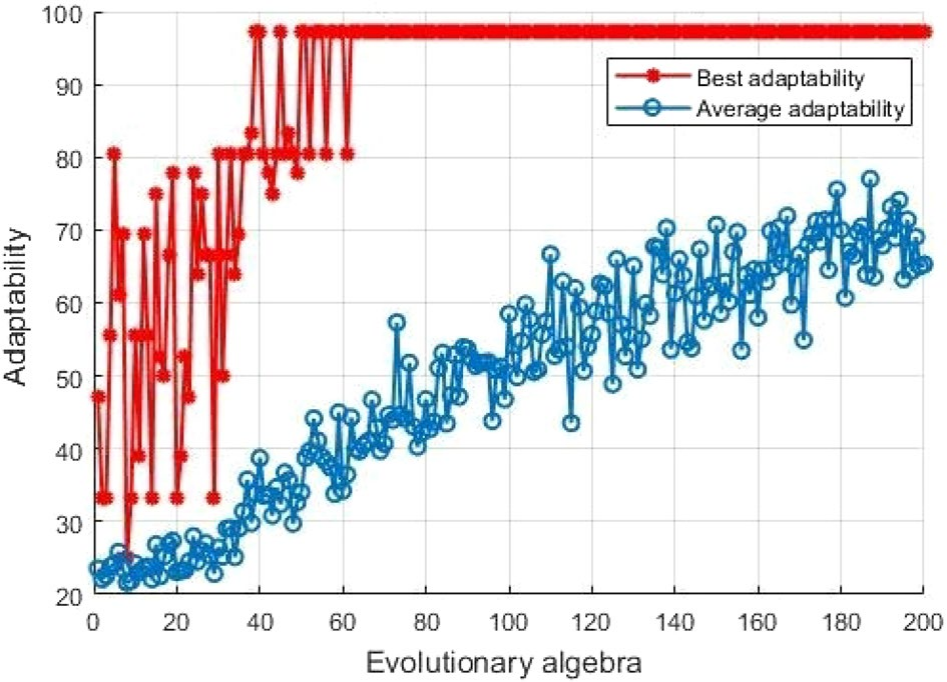
The result of QGA optimizing SVM parameters.

In the paper, QGA-SVM is used for fault identification. The experimental results show that the method can effectively diagnose building electrical system faults compared with traditional SVM, GA-SVM, and 1DCNN. As shown in Table 5 and Fig. 11, when the preprocessing methods are the same, the accuracy of the fault classification method proposed is higher than other fault classification methods, which verifies the effectiveness of the method. Figure 12 shows the results of fault diagnosis and identification by QGA-SVM. It can be seen from the figure that when the evolutionary algebra is greater than 60, its optimal fitness does not change.

The parameters in the experiment set the maximum evolutionary generation to 200, the maximum number of populations to 40, the variation range of *C* (0, 100], the variation range of $\gamma$ (0, 1000], and the number of SVM cross-validation is 5. The experimental results show that the optimal *C* and $\gamma$ parameters obtained by QGA optimizing SVM are 25.0702 and 0.01163, respectively. Compared with traditional feature extraction methods EMD, LMD, SVD and traditional SVM, GA-SVM, and 1DCNN for fault classification, this method is more suitable for building electrical appliances. The model has good fault diagnosis and classification effect and high accuracy, up to 91.67$\%$.

#### Comparison with experimental methods in the literature

**Figure 13:**
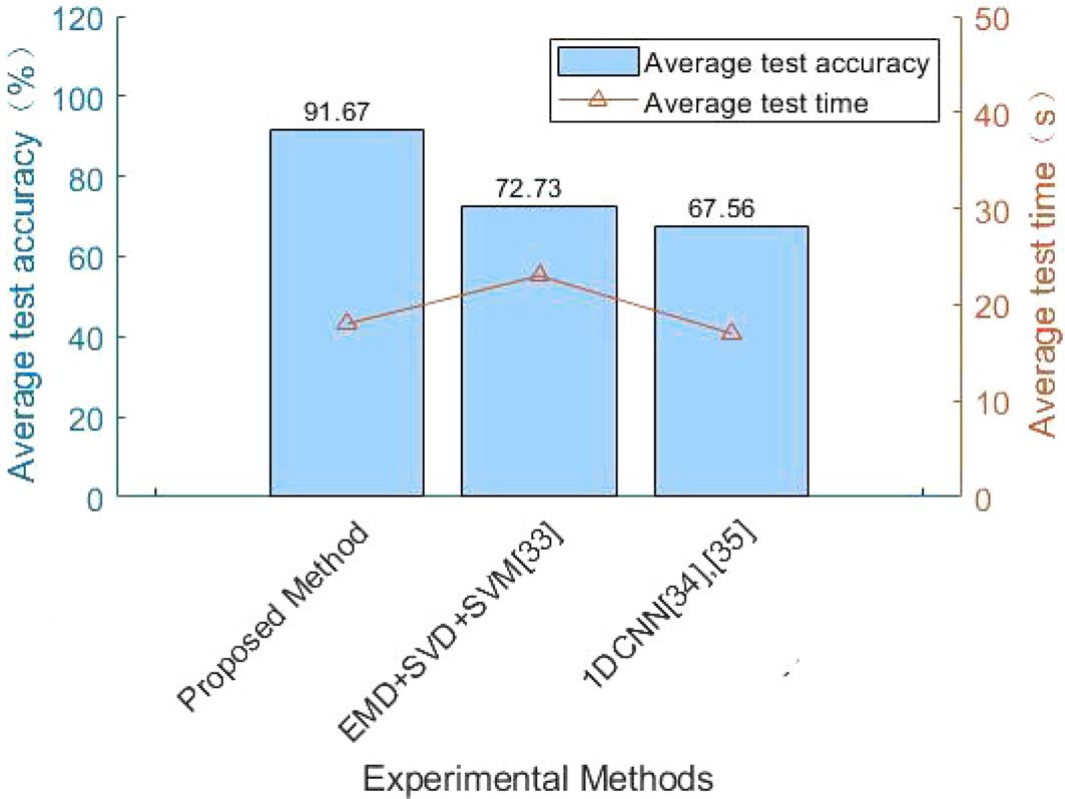
Comparison of the proposed method with other methods in the literature.

This paper selects some classic fault diagnosis methods of other papers to compare their performance, such as EMD+SVD+SVM^33^, 1DCNN^34,35^. And compared with the VMD+MDI+QGA-SVM method in this paper, the denoising effect, feature extraction effect and fault classification effect are relatively poor. And none of them can effectively identify the fault resistance signal of the building electrical system for fault classification. Therefore, the method of VMD+MDI+QGA-SVM is used for fault diagnosis in this paper. The experimental results are shown in Table 6 and Fig. 13, verifies the effectiveness of the method proposed in this paper.

## Discussions

The main work of this paper is devoted to data preprocessing and fault classification. It can be seen from Table 3 that the data preprocessing method VMD+MDI proposed has better effect and higher accuracy than EMD+MDI, LMD+MDI, to make the fault characteristics more obvious. The redundant and indistinct noise signal is filtered by VMD, and some different IMFs are decomposed. The appropriate number of IMFs can be selected by the similarity of the center frequency, and the fault features can be better expressed by extracting the MDI which is more sensitive to the fault characteristics.

As can be seen from Table 4, the difference in the number of decomposition modes and the proportion of data sets will have a big or small impact on the experimental results. The number of VMD decomposition modes adopted in this paper is 8 and the ratio of training set to test set is 3:1. In this case, the proposed QGA-SVM fault classification method has higher average test accuracy and better robustness, and the average test accuracy of fault classification can reach 91.67$\%$. It can be used for feature extraction and fault recognition and classification.

As can be seen from Table 5, the fault classification method QGA-SVM proposed has higher test accuracy and shorter running time than SVM, GA-SVM and 1DCNN. The QGA-SVM algorithm is set by QGA some parameters, such as the maximum evolutionary generation is 200, the maximum number of population is 40, the variation range of *C* (0, 100], the variation range of $\gamma$ (0, 1000], the number of SVM cross-validation is 5, further optimize the *C* and $\gamma$ parameters of SVM.

As can be seen from Table 6, the fault classification method QGA-SVM proposed has higher average test accuracy and better robustness than other fault classifications in the literature. It can perform feature extraction and fault identification and classification very well. Therefore, the method proposed can provide a good idea for fault diagnosis of building electrical systems in the future.

## Conclusions and future work

This paper proposed a new method for fault diagnosis of building electrical systems based on the combination of VMD-MDI and QGA-SVM. Firstly, the resistance fault signal is collected by the building electrical experimental platform MA2067 and the instrument Eurotest 61577. Secondly, the fault data is denoised by the VMD method, and there is almost no modal aliasing phenomenon compared with EMD, because VMD can select the number of modes by the similarity of frequencies. Thirdly, MDI is extracted for each decomposed mode. Finally, MDI with higher feature sensitivity is selected and input into the QGA-SVM model. The QGA-SVM model mainly selects the *C* and $\gamma$ parameters of the SVM through QGA optimization to diagnose and classify the faults of the building electrical system. The experimental results show that compared with EMD, LMD, SVD and SVM, 1DCNN, GA-SVM, the fault diagnosis method proposed is more robust and has better classification effect, with an accuracy rate of 91.67%. This paper only selects six faults in the three major categories for diagnosis and identification. The work to be done in the future is to use adaptive VMD to extract fault features and identify all inter-class and intra-class faults in the building electrical experiment platform. The diagnosis of composite faults is a future research direction.

## Data Availability

Inquiries regarding data availability should be directed to the second author. Its email address is 1721588525@qq.com
